# Fusobacterium Nucleatum in Colorectal Cancer: Relationship Among Immune Modulation, Potential Biomarkers and Therapeutic Implications

**DOI:** 10.3390/ijms26199710

**Published:** 2025-10-06

**Authors:** Dalila Incognito, Giuliana Ciappina, Claudia Gelsomino, Antonio Picone, Pierluigi Consolo, Alessandra Scano, Tindara Franchina, Nicola Maurea, Vincenzo Quagliariello, Salvatore Berretta, Alessandro Ottaiano, Massimiliano Berretta

**Affiliations:** 1 Medical Oncology Unit, Department of Human Pathology “G. Barresi”, School of Specialization in Medical Oncology, University of Messina, 98122 Messina, Italy; dalilaincognito19@gmail.com (D.I.); claudiagelsomino94@gmail.com (C.G.); 2 Section of Experimental Medicine, Department of Medical Sciences, University of Ferrara, 44121 Ferrara, Italy; giuliana.ciappina@unife.it; 3 Department of Clinical and Experimental Medicine, University of Messina, 98122 Messina, Italy; antonio.picone@polime.it; 4 Unit of Digestive Endoscopy, G. Martino University Hospital, University of Messina, 98125 Messina, Italy; pconsolo@unime.it; 5 Department of Surgical Sciences, University of Cagliari, 09124 Cagliari, Italy; alessandra.scano77@unica.it; 6 Department of Human Pathology of Adulthood and Childhood “G. Barresi”, University of Messina, 98122 Messina, Italy; tindara.franchina@polime.it; 7 Division of Cardiology, IRCCS Istituto Nazionale Tumori “Fondazione G. Pascale”, 80131 Naples, Italy; n.maurea@istitutotumori.na.it (N.M.); quagliariello.enzo@gmail.com (V.Q.); 8 Department of Human Pathology “G. Barresi”, G. Martino University Hospital, University of Messina, 98122 Messina, Italy; salvatore.berretta@studenti.unime.it; 9 Istituto Nazionale Tumori di Napoli, IRCCS “G. Pascale”, Via M. Semmola, 80131 Naples, Italy; ale.otto@libero.it; 10 Division of Medical Oncology, G. Martino University Hospital, University of Messina, 98122 Messina, Italy

**Keywords:** *Fusobacterium nucleatum*, colorectal neoplasm, immune checkpoint inhibitors, immunotherapy, biomarkers, drug resistance

## Abstract

*Fusobacterium nucleatum* (*Fn*) has been increasingly recognized as a crucial mediator of colorectal cancer (CRC) biology, particularly in microsatellite-stable (MSS) tumors, where immune checkpoint inhibitors (ICIs) have shown limited efficacy. Rather than representing a passive microbial passenger, *Fn* actively shapes tumor behavior by adhering to epithelial cells, activating oncogenic signaling, and promoting epithelial–mesenchymal transition (EMT). At the same time, it remodels the tumor microenvironment, driving immune suppression through inhibitory receptor engagement, accumulation of myeloid-derived cells, and metabolic reprogramming of tumor-associated macrophages. These mechanisms converge to impair cytotoxic immunity and contribute to both intrinsic and acquired resistance to ICIs. Beyond immune escape, *Fn* interferes with conventional chemotherapy by sustaining autophagy and blocking ferroptosis, thereby linking microbial colonization to multidrug resistance. Most of these mechanisms derive from preclinical in vitro and in vivo models, where causal relationships can be inferred. In contrast, human data are mainly observational and provide correlative evidence without proving causality. No interventional clinical studies directly targeting *Fn* have yet been conducted. Its enrichment across the adenoma–carcinoma sequence and consistent detection in both tumor and fecal samples highlight its potential as a biomarker for early detection and patient stratification. Importantly, multidimensional stool assays that integrate microbial, genetic, and epigenetic markers are emerging as promising non-invasive tools for CRC screening. Therapeutic strategies targeting *Fn* are also under exploration, ranging from antibiotics and bacteriophages to multifunctional nanodrugs, dietary modulation, and natural microbiota-derived products. These approaches may not only reduce microbial burden but also restore immune competence and enhance the efficacy of immunotherapy in MSS CRC. Altogether, current evidence positions *Fn* at the intersection of microbial dysbiosis, tumor progression, and therapy resistance. A deeper understanding of its pathogenic role may support the integration of microbial profiling into precision oncology frameworks, paving the way for innovative diagnostic and therapeutic strategies in CRC.

## 1. Introduction

Colorectal cancer (CRC) remains one of the most prevalent and lethal malignancies worldwide, with its incidence steadily rising, especially among individuals under the age of 50 [1]. The median age of diagnosis of CRC is 70–72 years, while approximately 19–22% already have metastatic disease at diagnosis [2]. According to the ASCO guidelines, treatment selection in metastatic CRC is based on microsatellite stability and mismatch repair status, RAS mutation status, and the location of the primary tumor (left versus right colon). In patients with previously untreated and initially unresectable disease, a doublet chemotherapy regimen, or when appropriate a triplet regimen, combined with an anti-VEGF agent, is recommended as first-line therapy [3].

In the presence of microsatellite instability-high (MSI-H) or deficient mismatch repair (dMMR), immunotherapy with anti-PD-1 or anti-CTLA-4 agents should be considered. For patients with microsatellite-stable (MSS) or proficient mismatch repair tumors and RAS wild-type status, treatment also depends on tumor sidedness. Left-sided tumors should be treated with chemotherapy plus anti-EGFR agents, whereas right-sided tumors are better managed with chemotherapy combined with anti-VEGF therapy [2].

Most metastatic CRC cases are MSS and exhibit poor responsiveness to ICIs, highlighting the urgent need to identify novel mechanisms of resistance and predictive biomarkers to improve therapeutic outcomes [4]. In recent years, the gut microbiota has emerged as a critical regulator of antitumor immunity, influencing both local and systemic immune responses and potentially modulating the efficacy of cancer immunotherapies [5].

Fusobacterium nucleatum (*Fn*) is one of the most frequently detected microbial species in CRC tissues and has been implicated in immune suppression, tumor progression, and poor prognosis [6]. *Fn* has been associated with resistance to ICIs in CRC, partly through the suppression of cytotoxic lymphocyte activity and disruption of interferon signaling pathways [4].

Recent evidence further indicates that *Fn*-derived metabolites, such as succinic acid, may impair the cGAS–STING–IFN-β axis, thereby reducing dendritic cell activation and compromising the efficacy of PD-1 blockade [3]. This suggests that microbial metabolism represents an additional layer of resistance beyond canonical immune checkpoint pathways. In parallel, clinical guidelines continue to emphasize molecular stratification for treatment personalization, yet they do not currently account for microbiota-driven resistance mechanisms. Integrating microbial signatures such as *Fn* into existing frameworks could enhance patient stratification and refine therapeutic decision-making, especially for MSS CRC where therapeutic innovation is most needed [2].

Given the increasing interest in microbiota-driven mechanisms of resistance, a focused review on *Fn* is timely and necessary. Summarising current knowledge may support the development of new strategies to overcome immune resistance in MSS CRC. This review critically examines current evidence on the role of *Fn* in shaping immune dynamics within the CRC microenvironment, with a focus on its impact on immunotherapy efficacy, and outlines the rationale for future clinical investigation in this setting.

## 2. Materials and Methods

**Figure 1:**
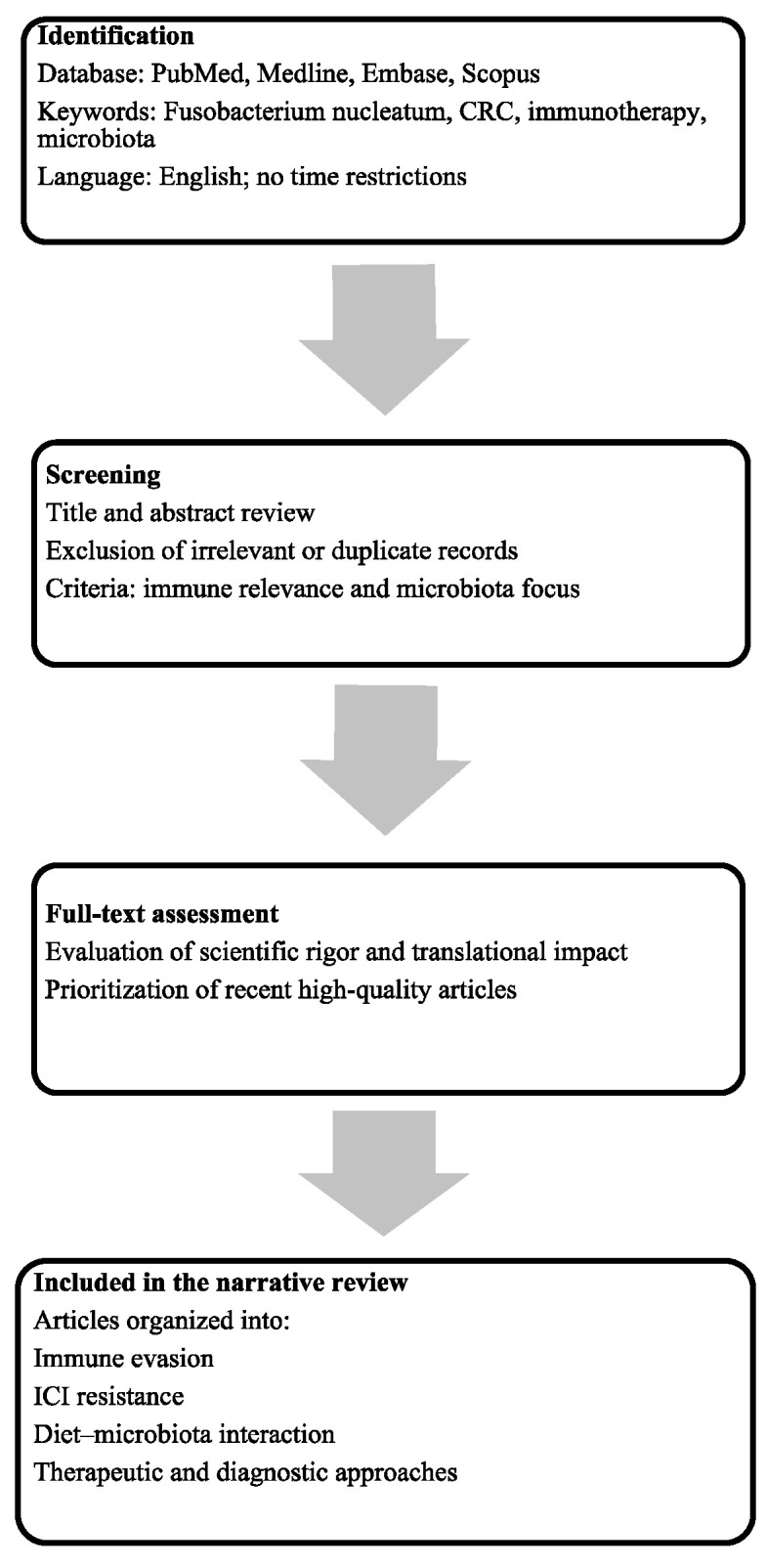
Flow diagram outlining the article selection process. Relevant literature was identified through structured database (all databases accessed on 30 August 2025) searches, screened for thematic relevance, and included based on scientific rigor and contribution to the review focus.

This narrative review was conducted to explore the role of Fn in modulating immune response and resistance to immunotherapy in metastatic CRC. The research was conducted using the PubMed, Medline, Embase, and Scopus (all accessed on 30 August 2025) from database inception to 30 August 2025, using controlled vocabulary and free-text terms, including the MeSH headings: “Fusobacterium nucleatum” (MeSH Unique ID: D016967), “colorectal neoplasm” (MeSH Unique ID: D015179), “neoplasm metastasis” (MeSH Unique ID: D009362), “immune checkpoint inhibitors” (MeSH Unique ID: D000082082), and “immunotherapy” (MeSH Unique ID: D007167). All types of peer-reviewed articles written in English were considered, including preclinical studies, clinical studies, reviews, and meta-analyses. Articles were excluded if they were unrelated to the topic of interest or lacked relevance to the role of *Fn* in CRC. The selection process prioritized recent publications and studies providing mechanistic or translational insights into *Fn*-induced immunotherapy resistance. References from key review articles were also screened to identify additional relevant sources. A summary of the literature selection process is presented in Figure 1.

## 3. Microbiota and Cancer

The human microbiome is composed of a large consortium of microorganisms, including bacteria, viruses, archaea, and fungi, that inhabit various anatomical sites of the human body. The gastrointestinal tract hosts the largest and most diverse microbial population, which plays essential roles in nutrient metabolism, immune modulation, and maintenance of mucosal integrity [5,6]. Although the bacterial component is the most widely studied, recent findings highlight the importance of fungal and viral communities in influencing host-microbiota interactions and systemic pathophysiology [7]. Alterations in the composition or function of the microbiota, commonly known as dysbiosis, have been implicated in the development of various diseases, including cancer, through mechanisms such as chronic inflammation, genotoxicity, and immune evasion. Chronic dysbiosis can foster a pro-inflammatory microenvironment characterized by persistent cytokine release, oxidative stress, and impairment of DNA repair pathways, thereby creating favorable conditions for tumor initiation and progression [5]. Microbiota-induced carcinogenesis is not limited to the gastrointestinal tract, but significant alterations in the mammary microbiota have been observed in breast cancer patients, with marked differences between benign and malignant breast lesions. Microbial dysbiosis in other body sites also contributes to the development of breast cancer. For example, periodontitis, caused by oral dysbiosis, has been associated with an increased risk of breast cancer [8]. We have also seen that in cervical cancer, changes in cervicovaginal microbial diversity, particularly reductions in *Lactobacillus* species, have been associated with persistent human papillomavirus infection and increased cancer risk [9,10]. Specifically, cervicovaginal communities depleted of *Lactobacillus crispatus* and dominated by *Lactobacillus iners* or anaerobic bacteria have been linked to higher genital inflammation, HPV persistence, and progression to intraepithelial neoplasia, highlighting the protective role of a *Lactobacillus*-dominant environment [9]. These observations highlight the broader oncological relevance of microbial ecosystems and their potential diagnostic and therapeutic applications in multiple organ systems [11]. Indeed, the integration of microbiome signatures into cancer risk assessment and treatment stratification is increasingly viewed as a promising avenue for precision oncology [5].

## 4. Taxonomy and Subspecies Diversity of Fn

*Fn* has long been divided into four major subspecies (nucleatum, animalis, polymorphum, and vincentii), which were historically regarded as functionally interchangeable. However, recent genomic and phylogenetic analyses have provided compelling evidence that these subspecies represent distinct species rather than minor variants. Average nucleotide identity (ANI) values between subspecies are below the 95% threshold commonly accepted for species delineation, further supporting this reclassification [12]. Historically referred to as *Fn subsp. animalis* (*Fna*), this taxon has been reclassified as the distinct species *Fusobacterium animalis* (*F. animalis*) according to recent genomic and phylogenetic analyses. Among them, *F. animalis* is consistently enriched in CRC tissues and shows the strongest associations with patient outcome [13]. Metagenomic studies have identified two major clades within *F. animalis*, designated C1 and C2, with the latter dominating biofilm-positive CRC tumors [13]. Subsequent investigations revealed that the *F. animalis* C1 cluster actually corresponds to a separate species, *Fusobacterium watanabei*, while a novel species named *Fusobacterium paranimalis* has also been recently described [14]. This taxonomic revision has important clinical implications, as not all members of the *Fn* group display the same virulence potential. The predominance of *F. animalis*, especially the *F. animalis* C2 clade, in CRC underscores the need for diagnostic and therapeutic strategies that specifically target the relevant pathogenic taxa rather than treating *Fn* as a homogeneous entity [15].

## 5. Microbial Colonization and Tissue Tropism

*Fn* is consistently detected at higher levels in CRC tissue than in adjacent normal mucosa and has been identified in both fecal material and archived tumor specimens from patients with metastatic CRC [4]. The adhesion of *Fn* to colorectal epithelial cells is primarily mediated by the outer membrane protein FadA, which binds to E-cadherin and activates β-catenin signaling, thereby facilitating bacterial internalization and promoting tumorigenesis [5]. In addition to FadA, other adhesins play essential and strain-specific roles in colonization. The large autotransporter protein RadD is a major adhesin that mediates coaggregation with partner bacteria and contributes to biofilm architecture; its regulation by the lipoprotein FAD-I highlights a complex network controlling interspecies interactions. Moreover, RadD has been implicated in lymphocyte cell death, suggesting a role in modulating immune surveillance [16]. Together with RadD, the Fap2 adhesin enables binding to tumor-associated glycans and interacts with TIGIT on NK and T cells, thereby promoting immune evasion within the tumor microenvironment. These findings indicate that Fap2 and RadD, rather than FadA alone, may be particularly relevant to the oncogenic and immunomodulatory effects of this bacterium [15]. *Fn* can also cross the intestinal epithelial barrier by altering tight junction integrity, contributing to a local inflammatory environment that favors tissue invasion and intratumoral persistence [7].

**Figure 2:**
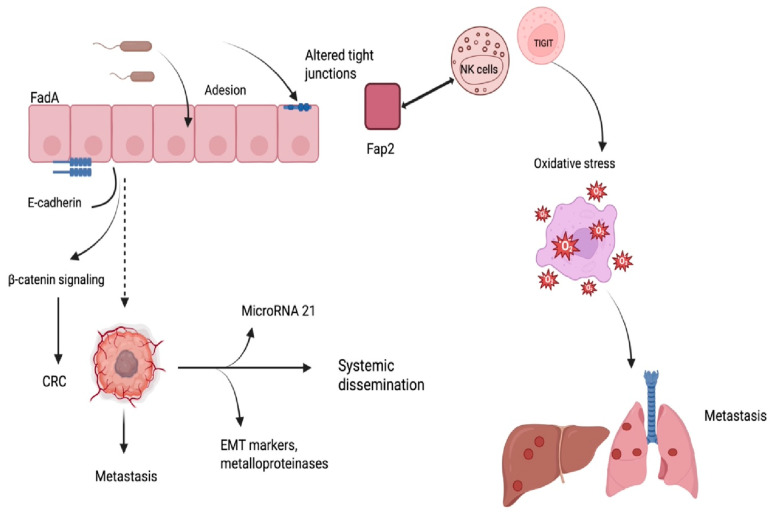
Mechanisms of Fn colonization in CRC, encompassing epithelial adhesion via FadA–E-cadherin interaction, TIGIT-mediated immune suppression, and promotion of metastatic dissemination. Abbreviations: CRC, colorectal cancer; EMT, Epithelial–Mesenchymal Transition; FadA, Fusobacterium adhesin A; Fap2, Fusobacterium outer-membrane autotransporter 2; Fn, *Fusobacterium nuclatum*; NK, natural killer; TIGIT, T-cell immunoreceptor with Ig and ITIM domains.

*Fn* has been shown to promote CRC progression through the activation of oncogenic pathways and the suppression of host defense mechanisms. The bacterium adheres to epithelial cells and activates the E-cadherin/β-catenin signaling axis, leading to the transcription of genes involved in cell proliferation and tumor development [16]. In addition, the bacterium enhances the expression of microRNA-21, which promotes tumor cell invasion and proliferation by targeting tumor suppressor genes [6]. Experimental models have demonstrated that exposure to *Fn* increases the invasive potential of CRC cells by inducing epithelial-to-mesenchymal transition (EMT) markers and metalloproteinases, facilitating extracellular matrix degradation and tumor spread [7,17]. Furthermore, EMT is a well-recognized process in various malignancies associated with peritoneal dissemination and therapeutic resistance [18]. In addition, oxidative stress induced by the tumor microenvironment may drive *Fn* o transition from a commensal to a pathogenic phenotype, enhancing its ability to contribute to carcinogenesis [19] (Figure 2). Recent studies demonstrated that Fn colonization of CRC tissue is facilitated by recognition of tumor-associated glycans, particularly Gal-GalNAc, through the Fap2 adhesin, allowing selective bacterial accumulation in malignant cells [16]. Beyond CRC, *Fn* dissemination to distant organs has been documented, and circulating bacteria may seed metastatic sites through hematogenous spread, with evidence of identical strains detected in both primary and liver metastases [6]. *Fn* has also been shown to potentiate chemoresistance by modulating cell death pathways. In particular, it induces oxaliplatin resistance through activation of the E-cadherin/β-catenin/TCF4 axis and upregulation of GPX4, thereby suppressing ferroptosis, a form of regulated cell death critical for chemotherapy efficacy [16]. Beyond its local effects, *Fn* is capable of entering the bloodstream and colonizing distant organs, where it may contribute to metastatic dissemination. Its detection in metastatic lesions of colorectal origin suggests a possible role in the establishment and progression of secondary tumors [6].

## 6. Immune Modulation and Tumor Immune Evasion

Within the CRC microenvironment, experimental studies have shown that *Fn* can exert immunomodulatory effects contributing to tumor immune escape and reduced sensitivity to therapy. Through its outer membrane protein Fap2, it binds to the inhibitory receptor TIGIT on T and natural killer (NK) cells, dampening their cytotoxic functions [16]. In parallel, engagement of CEACAM1 further compromises NK cell activity, weakening antitumor immune responses [16,17]. The bacterium has been reported in preclinical models to promote the accumulation of immunosuppressive cell populations, including myeloid-derived suppressor cells (MDSCs) and tumor-associated macrophages (TAMs), which inhibit T-cell proliferation and interfere with ICIs’ efficacy [17]. Tumors colonized by this microorganism show increased MDSC infiltration and impaired interferon signaling, correlating with attenuated responses to immunotherapy [18]. In murine models, *Fn* enrichment significantly increased MDSC and TAM recruitment, creating an immunosuppressive tumor microenvironment that directly impaired immune checkpoint blockade efficacy [17]. In murine models, *Fn* enrichment has been associated with increased MDSC infiltration and impaired interferon signaling, correlating with attenuated responses to immunotherapy [17]. Additionally, T-cell apoptosis and suppression of adaptive antitumor immunity have been observed in experimental systems through activation of the Wnt/β-catenin pathway. These changes are accompanied by upregulation of proinflammatory cytokines, such as IL-8 and CXCL1, which recruit neutrophils and drive their polarization toward tumor-supportive phenotypes [6].

Finally, immune evasion is reinforced by the bacterial metabolite succinic acid, which impairs the cGAS-STING-IFN-β signaling cascade, thereby compromising dendritic cell activation and antigen presentation in the context of PD-1 blockade [4,17]. Beyond immune evasion, preclinical evidence suggests that Fn may contribute to metastatic progression by reshaping the tumor microenvironment through immunomodulatory mechanisms. Specifically, infection with *Fn* induces CCL20 expression via NF-κB activation and downregulation of miR-1322, leading to macrophage recruitment and M2 polarization, a phenotype associated with tumor-promoting immunosuppression. This miR-1322/CCL20 axis not only enhances M2 macrophage polarization but also fosters pre-metastatic niche formation, thereby linking immune evasion with metastatic dissemination [19]. These M2-like macrophages enhance the migratory and invasive behavior of CRC cells in both in vitro and in vivo models. Moreover, clinical observations reporting the detection of Fn in metastatic lesions, coupled with reduced CD8^+^ and FOXP3^+^ lymphocyte infiltration, suggest an association with the establishment of immunologically permissive pre-metastatic niches [19,20]. These mechanisms are summarized in Table 1.

The contribution of *Fn* to immune escape is particularly relevant in MSI tumors, where microbial genotoxins exacerbate genomic instability and promote upregulation of immune checkpoints such as PD-1 and CTLA-4, ultimately dampening the expected benefit of immunotherapy [18].

## 7. Clinical Implications and Immunotherapy Resistance

While ICIs have improved outcomes in several malignancies, their efficacy in CRC remains limited to a minority of patients with MSI-H or dMMR tumors, accounting for only approximately 5% of metastatic cases. Given the limited effectiveness of ICIs in MSS CRC, identifying novel predictive biomarkers remains a clinical priority. In this context, emerging evidence highlights the role of gut microbiota composition, which can be significantly influenced by dietary habits, as a potential modulator of immune responsiveness and immunotherapy efficacy [21].

**Figure 3:**
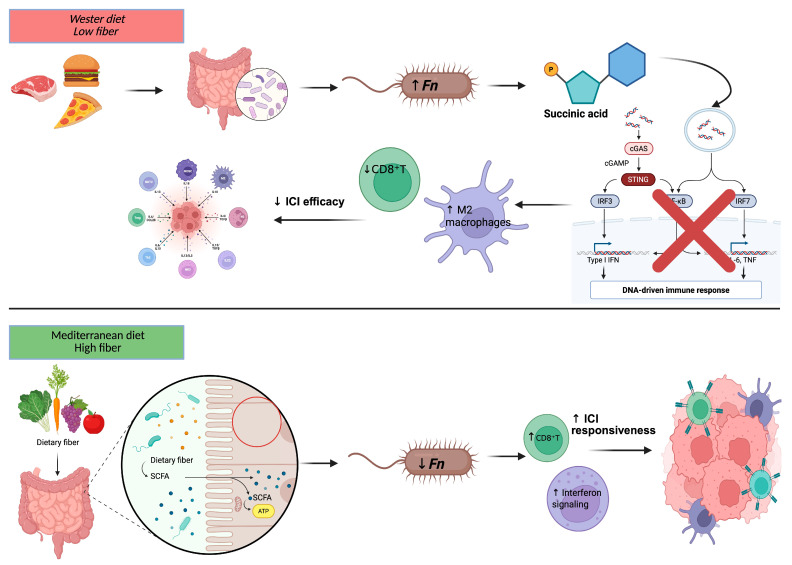
Dietary modulation of microbiota and immune landscape in CRC. Abbreviations: CD8, cluster of differentiation 8; CRC, colorectal cancer; Fn, *Fusobacterium nucleatum*; ICIs, immune checkpoint inhibitors; ↑/↓ = increase/decrease.

Dietary composition plays a central role in shaping the gut microbiota and, consequently, modulating the interplay between *Fn*, the immune system, and CRC progression. Epidemiological studies have reported associations between dietary patterns rich in industrially processed foods and low in fiber and an increased risk of CRC subtypes enriched in *Fn* and other oncogenic bacteria, such as pks+ *Escherichia coli* and *enterotoxigenic Bacteroides fragilis* [22,23]. These microbial subtypes are typically associated with impaired host immune responses and reduced sensitivity to immune checkpoint blockade. High consumption of red and processed meat has been associated with dysbiosis and enrichment of pro-inflammatory taxa such as *Fn*, possibly mediated through the formation of mutagenic compounds, including N-nitroso compounds and heterocyclic amines, and by promoting chronic mucosal inflammation [24]. In contrast, fiber-rich diets, particularly those containing prebiotic substrates such as polydextrose and fibersol-2, have been shown in experimental and observational studies to reduce the pathogenic potential of *Fn* by enhancing butyrate production capacity, thereby suppressing tumor cell proliferation and modulating inflammatory gene expression [25]. The Mediterranean diet, characterized by a high intake of plant-based foods and olive oil, has been linked to a lower incidence of CRC, and its protective effect may be partly mediated by modulation of gut microbiota and attenuation of *Fn*-related pro-carcinogenic signatures [26,27]. Significantly, microbiota-mediated effects on the tumor immune environment have been suggested to influence not only cancer progression but also the response to immunotherapy [28,29]. Specific dietary components and microbial-derived metabolites, such as short-chain fatty acids, have been reported in preclinical models to enhance antitumor immune surveillance and to improve the efficacy of ICIs [27,30]. In addition to its immunomodulatory effects, *Fn* has been shown in both preclinical models and translational studies to induce resistance to oxaliplatin and 5-fluorouracil in CRC by activating the TLR4–MYD88 signaling axis, which promotes autophagy and inhibits apoptosis, ultimately reducing chemotherapy efficacy [31,32]. Altogether, the available evidence supports the hypothesis that dietary modulation of the gut microbiota may represent a strategy to reduce intratumoral *Fn* burden, reshape the immune landscape, and potentially enhance immunotherapy responsiveness in MSS CRC, although prospective validation in clinical trials is still required (Figure 3).

Furthermore, recent studies have highlighted that *Fn* may act as a modulator of immunotherapy efficacy in CRC. Observational data indicate that colonization of the CRC microenvironment by *Fn* is associated with adverse prognostic features and reduced responsiveness to ICIs in MSS tumors [6]. Moreover, *Fn*-derived outer membrane vesicles have been shown in preclinical studies to reprogram tryptophan metabolism within tumor-associated macrophages, leading to the production of immunosuppressive kynurenine and suppression of antitumor immunity [28]. Single-cell RNA sequencing analyses from translational studies further suggest that this microorganism can modulate immune cell composition and checkpoint expression profiles. In *Fn* positive CRC, reduced CD8+ T-cell infiltration and altered interferon signaling have been reported, correlating with diminished response to PD-L1 blockade [29].

Additionally, microbial metabolites such as succinic acid have been implicated in preclinical models in suppressing the cGAS–STING pathway, a key driver of type I interferon responses necessary for dendritic cell activation and T-cell priming during ICIs therapy [6]. Based on these findings, *Fn* has been proposed as a potential prognostic marker and a modifiable factor influencing ICIs’ efficacy, but this remains to be demonstrated in prospective clinical trials. Preclinical evidence suggests that modulating the microbiota composition or selectively targeting *Fn* may restore antitumor immunity and potentially improve immunotherapy outcomes [30].

## 8. Therapeutic Targeting Strategies

**Figure 4:**
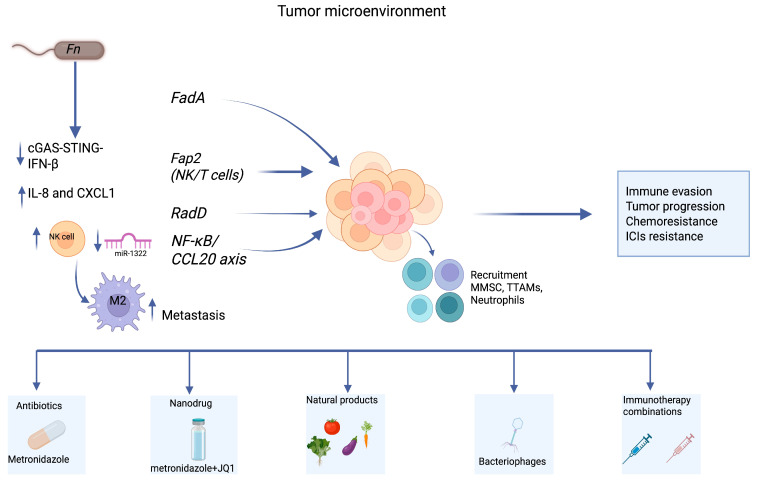
Schematic overview of *Fn* mechanisms in CRC and related therapeutic strategies. Abbreviations: cGAS, cyclic GMP–AMP synthase; CXCL1, C-X-C motif chemokine ligand 1; FadA, Fusobacterium adhesin A; Fap2, Fusobacterium outer-membrane autotransporter 2; Fn, *Fusobacterium nucleatum*; IFN-β, interferon-beta; IL-8, interleukin-8; STING, stimulator of interferon genes. ↑/↓ = increase/decrease.

Given the involvement of *Fn* in CRC progression and resistance to therapy, multiple strategies have been explored to target this microorganism and its associated pathogenic effects. Preclinical models have demonstrated that treatment with metronidazole significantly reduces intratumoral bacterial load and tumor burden, supporting the use of antibiotics as a direct approach to limit microbial-driven oncogenesis [7]. In addition to antibiotics, novel delivery systems such as multifunctional nanodrugs have shown promise in addressing both chemoresistance and immunosuppression associated with *Fn*. For example, co-encapsulation of metronidazole with the BET inhibitor JQ1 in a nanocarrier enhanced oxaliplatin efficacy and promoted CD8^+^ T cell infiltration in murine models [31]. Recent studies have also shown that outer membrane vesicles released by *Fn* modulate tryptophan metabolism in tumor-associated macrophages, enhancing kynurenine production and promoting resistance to PD-1 blockade. This highlights the need for therapeutic approaches aimed not only at bacterial eradication but also at neutralizing vesicle-mediated immunosuppression [28]. Other approaches include the use of bacteriophages, which are capable of selectively lysing *Fn* without disrupting commensal flora, and small-molecule inhibitors that interfere with microbial adhesion or virulence factors. Combination strategies targeting both the bacterium and the tumor microenvironment may be particularly effective in restoring immune competence and enhancing responsiveness to immunotherapy [33] (Figure 4). In parallel, natural products such as pectin, inulin, anthocyanins, and ginsenosides have demonstrated the ability to reshape gut microbiota composition and enhance antitumor immunity. By increasing beneficial taxa (e.g., *Bifidobacterium*, *Akkermansia*) and boosting short-chain fatty acid production, these compounds can improve CD8^+^ T-cell infiltration and sensitize tumors to checkpoint blockade [30]. Interestingly, specific microbial components or attenuated strains of *Fn* have also been investigated as adjuvants for immunotherapy. In selected models, the bacterium augmented PD-L1 blockade efficacy, possibly by activating innate immunity pathways. This paradoxical effect suggests that under controlled conditions, microbial modulation could support rather than hinder therapeutic outcomes. Finally, oxidative stress has been implicated in promoting the shift of *Fn* from a commensal to a pathogenic state. The use of antioxidants to preserve redox balance may therefore offer a supportive approach to limit its pro-tumorigenic effects [34]. An overview of these approaches is provided in Table 2.

## 9. Biomarker Value and Diagnostic Applications

**Figure 5:**
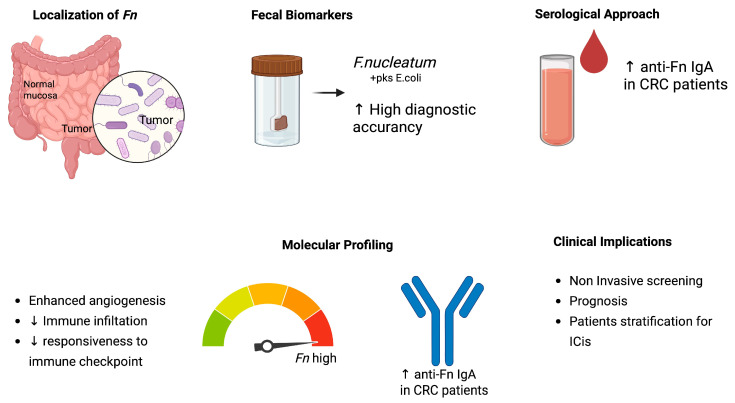
Diagnostic and prognostic relevance of *Fn* in CRC, illustrating detection modalities and associations with distinct tumor immunophenotypes. Abbreviations: CRC, colorectal cancer; Fn, *Fusobacterium nucleatum*; ICIs, immune checkpoint inhibitors; ↑/↓ = increase/decrease.

The growing evidence of *Fn* enrichment in CRC tissues compared to adjacent normal mucosa has highlighted its potential as a microbial biomarker for early detection and prognosis. In a large-scale meta-analysis, intestinal *Fn* demonstrated a pooled sensitivity of 0.72 and specificity of 0.76 for CRC detection, supporting its relevance as a non-invasive biomarker candidate [35]. Fecal detection of *Fn*, particularly when combined with other pathogens such as *pks+ Escherichia coli*, showed high diagnostic accuracy and was significantly more prevalent in patients with adenomas or carcinoma compared to healthy controls [36,37]. Serological approaches have also been investigated, with one study demonstrating that anti-*Fn* IgA levels were significantly elevated in CRC patients compared to controls, offering promise for developing blood-based diagnostic assays [24]. From a molecular perspective, *Fn* abundance has been associated with distinct tumor immunophenotypes. Transcriptomic analyses revealed that *Fn*-high CRC tumors exhibited enhanced angiogenesis-related gene expression, correlating with poorer immune infiltration and reduced responsiveness to immune checkpoint blockade [38] (Figure 5).

Furthermore, integrating microbiome signatures into predictive frameworks has emerged as a feasible tool for stratifying patients who may benefit from tailored interventions. Given its dual role in both tumorigenesis and immune modulation, *Fn* profiling could be incorporated into precision oncology approaches, not only for diagnostic purposes but also for treatment planning [39]. Finally, the sustained effort to refine detection methods for *Fn*, including quantitative PCR and next-generation sequencing, aims to enhance diagnostic sensitivity while maintaining clinical applicability [7].

*Fn* plays an active role in CRC progression, immune modulation, and immunotherapy to immunotherapy, particularly in MSS disease. Despite compelling preclinical and translational data, its integration into clinical practice remains limited. Current evidence underscores the need to move beyond observational associations and define the clinical significance of *Fn* through well-designed prospective studies.

## 10. Fecal Detection of Fn

It is important to distinguish between luminal and mucosal microbiota, as *Fn* is rarely detected in the healthy stool microbiome but is consistently enriched in colorectal mucosa, particularly in tumor-associated sites [40]. Studies have shown that the presence of *Fn* in stool reflects translocation from the oral cavity and subsequent colonization of the intestinal mucosa rather than stable membership of the luminal community [41]. Therefore, fecal detection of *Fn* should be interpreted as an indirect marker of mucosal colonization and biofilm formation in the colorectum, rather than evidence of its role as a commensal organism in the gut lumen [42,43].

The detection of *Fn* in stool has been investigated as a non-invasive strategy for identifying early stages of colorectal carcinogenesis [44]. The relative abundance of this microorganism was found to progressively increase from healthy individuals to patients with adenomas and overt CRC, suggesting a gradual enrichment along the adenoma-carcinoma sequence [39,45]. Its presence in fecal material reflects microbial dysbiosis associated with neoplastic transformation and has been proposed as a surrogate marker of tumor-associated alterations in the gut ecosystem [46]. *Fn* detection has also been integrated into broader diagnostic panels that include DNA methylation and mutation analysis, with the aim of improving the overall performance of stool-based screening approaches [47]. Recent findings have demonstrated that *Fn* levels in fecal samples correlate with systemic immune alterations, particularly the abundance of circulating neutrophil extracellular traps (NETs), which can promote angiogenesis, EMT, and metastatic dissemination. This suggests that fecal detection of *Fn* may also reflect inflammatory pathways relevant to tumor progression [48]. Despite this potential, recent studies have highlighted important limitations in the interpretation of fecal *Fn* levels. The association between this bacterium and CRC may be influenced by factors unrelated to tumor biology, including intestinal inflammation, host metabolic status, and technical aspects of stool processing [46,49]. In particular, pre-analytical variables play a major role: DNA extraction procedures are responsible for much of the variability observed across studies, while storage conditions and preservation methods can alter microbial profiles and affect the quantification of *Fn*. Moreover, differences in assay design such as strain-targeted qPCR, digital PCR, or shotgun metagenomics further impact sensitivity and reproducibility [50]. These observations underline the necessity of cautious evaluation when considering *Fn* as a fecal biomarker, particularly in the absence of inflammation-adjusted analytical models. A growing number of studies suggest that combining fecal *Fn* detection with other molecular features, such as stool DNA mutations or epigenetic alterations, may enhance the robustness of non-invasive screening approaches. Rather than focusing on a single microbial marker, multidimensional assays appear to provide a more reliable representation of the complex tumor-associated microbiome [40,51]. Moreover, the heterogeneity of results across populations and methodologies suggests that *Fn*, while promising, should not be considered an isolated diagnostic indicator [47,52]. Nevertheless, its inclusion in multi-target assays and the potential for integration with additional host-derived parameters, such as inflammatory mediators or epigenetic markers, represent relevant directions for future research [45,53]. Overall, a combined evaluation of microbial, genetic, and host-derived factors may support the development of precision-oriented, stool-based testing strategies, thereby expanding the utility of non-invasive tools in CRC.

## 11. Evidence Gaps and Conflicting Findings

Despite the growing body of evidence linking *Fn* to CRC, clinical evidence regarding its prognostic and predictive value remains inconsistent. High tumor *Fn* DNA levels were associated with poorer overall survival in some cohorts [54], whereas other large studies did not confirm an independent prognostic effect once clinicopathological factors and adjuvant treatments were considered [54]. A recent meta-analysis further demonstrated that prognostic associations were retained only in studies analyzing fresh frozen tissue, but not in those using FFPE samples, highlighting the influence of biospecimen type and detection methodology [55]. Geographic variability adds another layer of complexity: stronger associations have been reported in Asian cohorts compared with Western populations, suggesting that diet, host genetics, and baseline microbiota may modulate the relationship between *Fn* and clinical outcomes [28,37]. Finally, it is important to recognize that causal relationships between *Fn* and disease progression have been demonstrated only in preclinical models, whereas human studies remain correlative and sometimes contradictory. This raises the unresolved question of whether *Fn* represents a true driver of CRC or rather a passenger enriched in the altered tumor microenvironment. Altogether, these discrepancies highlight the need for harmonized detection protocols, multicenter validation across diverse populations, and well-designed interventional trials to establish whether *Fn* can be reliably used as a prognostic or predictive biomarker and as a therapeutic target in CRC.

## 12. Future Directions: Ethical Considerations and Trial Design

The prospect of targeting *Fn* in CRC raises important translational and ethical issues. Interventions such as antibiotics, bacteriophages, engineered probiotics, or microbiota transplantation carry potential risks of disrupting commensal microbial communities, which may be particularly relevant in oncology patients with impaired immunity or altered gut homeostasis. Careful safety monitoring, informed consent, and long-term follow-up should therefore be integral components of clinical development.

Future clinical trials evaluating *Fn*-targeted approaches will need to adopt rigorous designs, including standardized methods for bacterial detection, stratification by molecular subgroups (e.g., MSI versus MSS), and clinically meaningful endpoints such as treatment response and survival. Multicenter collaboration and harmonization of protocols will be essential to ensure reproducibility and generalizability of results. Such efforts will be necessary to establish whether targeting *Fn* can be translated into safe and effective therapeutic strategies in CRC.

## 13. Conclusions

Evidence from experimental models indicates that *Fn* contributes to CRC progression, modulation of antitumor immunity, and resistance to ICIs, particularly in MSS tumors. These data derive mainly from in vitro and in vivo studies, where Fn has been shown to adhere to epithelial cells, activate oncogenic pathways, and induce an immunosuppressive microenvironment. In contrast, clinical studies have so far been observational, supporting a correlation between intratumoral or fecal *Fn* abundance and poor prognosis, but not proving causality.

*Fn* has therefore been proposed as a potential microbial biomarker for CRC and for predicting responsiveness to immunotherapy, although its clinical validity remains to be established in prospective trials. Longitudinal studies combining baseline quantification of *Fn* with immune profiling during therapy will be necessary to clarify whether bacterial detection represents a static marker of resistance or a dynamic and modifiable factor.

To date, no interventional clinical trials have demonstrated that strategies aimed at reducing *Fn* burden, such as antibiotics, bacteriophages, engineered probiotics, or dietary interventions, improve outcomes in CRC. These approaches remain at the preclinical or early translational stage and require rigorous clinical evaluation.

Altogether, a clearer understanding of *Fn*–host interactions, integrating mechanistic data with well-designed clinical studies, is essential to define its role as both a biomarker and a therapeutic target. The incorporation of microbial profiling into precision oncology frameworks may ultimately contribute to more refined patient stratification and improved therapeutic strategies in MSS CRC.

## Figures and Tables

**Table 1:** Key mechanisms and immune interactions of *Fusobacterium nucleatum* CRC. Abbreviations: CRC, Colorectal Cancer; DC, Dendritic Cell; EMT, Epithelial–Mesenchymal Transition; Fn, *Fusobacterium nucleatum*; IFN-β, Interferon-beta; IL, Interleukin; NETs, Neutrophil Extracellular Traps; NF-κB, nuclear factor kappa B; TAM, Tumor-Associated Macrophage; TCF4, Transcription Factor 4; TIGIT, T-cell immunoreceptor with Ig and ITIM domains. ↓ = decrease.

Mechanism	Molecular Pathway	Role in CRC	Evidence
FadA	E-cadherin/β-catenin/TCF4	Adhesion, internalization, oncogenic signaling	Preclinical
Fap2	Gal-GalNAc binding; TIGIT receptor	Tumor colonization, lymphocyte inhibition, immune evasion	Preclinical and human
RadD	Autotransporter protein	Biofilm formation, lymphocyte killing, immune modulation	Preclinical
*Fn*-derived succinic acid	cGAS–STING–IFN-β	Inhibits DC activation, T-cell priming; PD-1 resistance	Preclinical
miR-21	Upregulated by *Fn*	Invasion, proliferation, metastasis	Preclinical and clinical
NF-κB/CCL20	NF-κB activation; ↓ miR-1322	TAM recruitment, M2 polarization, metastatic niche	Preclinical
Cytokines/NETs	IL-8, CXCL1, NETs	Angiogenesis, EMT, metastasis	Preclinical and translational

**Table 2:** Therapeutic strategies targeting *Fn* in CRC. Abbreviations: CRC, colorectal cancer; FMT, Fecal Microbiota Transplantation; *Fn*, *Fusobacterium Nucleatum*; MYD88, Myeloid Differentiation Primary Response 88; PD-1, Programmed Cell Death Protein 1; PD-L1, Programmed Death-Ligand 1; SCFA, Short-Chain Fatty Acids; TLR4, Toll-Like Receptor 4; TIGIT, T-cell Immunoreceptor with Ig and ITIM domains. ↑/↓ = increase/decrease.

Strategy	Mechanism/Target	Preclinical Evidence	Clinical Status/ Limitations
Antibiotics (e.g., metronidazole)	Reduce intratumoral *Fn*; modulate TLR4–MYD88	Reduced *Fn* load and tumor growth in murine CRC	No CRC-specific trials; microbiota disruption risk
Bacteriophages	Selective *Fn* lysis	Effective killing in vitro	Early preclinical; no in vivo CRC data
Nanodrug delivery (e.g., metronidazole and JQ1)	Overcome chemoresistance and immunosuppression	Enhanced oxaliplatin efficacy, ↑ CD8^+^ T cells in mice	Preclinical only; no human data
Probiotics/FMT	Restore microbiota balance; ↓ Fn colonization	Reduced *Fn* in colitis/CRC models	Limited translational studies; safety unproven
Natural products (inulin, anthocyanins, ginsenosides)	Enhance SCFA-producers; immunomodulation; ↓ Fn inflammation	↑ CD8^+^ T-cell infiltration, ↓ tumor burden in models	Preclinical and observational; no randomized trials
Immune checkpoint modulation	Block *Fn* mediated TIGIT; reverse immunosuppression	Improved PD-1/PD-L1 blockade in mice	Conceptual; no targeted clinical trials

## Data Availability

Not applicable.
